# Reflection to enhance dental students´ awareness of and comfort with uncertainty – an experimental study

**DOI:** 10.1186/s12909-025-06645-6

**Published:** 2025-01-10

**Authors:** Joséphine Brodén, Helena Fransson, Niklas Vareman, Maria Pigg

**Affiliations:** 1 Department of Oral Biology, Faculty of Odontology, Malmö University, Malmö, Sweden; 2 Department of Dental Medicine, Karolinska Institutet, Huddinge, Sweden; 3 Department of Endodontics, Faculty of Odontology, Malmö University, Malmö, Sweden; 4 Department of Medical Ethics, Lund University, Lund, Sweden

**Keywords:** [Clinical] decision-making, Education, dental, Endodontics, Periapical periodontitis, Uncertainty

## Abstract

**Background:**

Uncertainty is present in many situations in dental practice, but must not prevent wise clinical decision-making. Dental education should acknowledge uncertainty and teach useful management strategies. This study explored if dental students are aware of, and comfortable with uncertainty. The aims were to (i) measure students’ comfort or discomfort with and awareness of uncertainty while conducting risk assessment, and (ii) investigate whether a reflection exercise makes the students more aware of, and comfortable with, uncertainty.

**Methods:**

In January 2021, final-year students (*n* = 51) were randomized to either a structured written reflection exercise (intervention) or to a control exercise. Five months later, in June, each group was assigned the other exercise (cross-over design; ensuring a sufficient sample). Students’ statements of uncertainty and comfort were gathered using a developed questionnaire before and after the exercises. The students were blinded to which of the exercises was the intervention. The exercises and questionnaire were administered in mandatory sessions on an internet-based learning platform, ensuring anonymity and informed consent. Potential carryover effects were mitigated by analyzing intervention exercise data from both groups but control exercise data only from the first group.

**Results:**

At baseline 80% (41/51) of the students stated feeling very uncertain, uncertain or neither certain nor uncertain about assessing the risk and 84% were comfortable or very comfortable with their ability to handle the situation, with no between-group differences. The majority, 57% (29/51) of the students stated that they thought an experienced colleague would feel certain or very certain. After the exercise in June, 36% (9/25) of the students exposed to the reflection exercise changed their statements on how certain they felt about their capacity to handle the case.

**Conclusions:**

The exercise did not affect the awareness of uncertainty and the students’ comfort with it, as the majority of students stated already feeling comfortable in their ability to handle the situation at baseline. However, the reflection exercise highlighted the students’ perception that experience is important in managing uncertainty. There is a need for further research to better understand students’ and teachers’ perception and attitudes to uncertainty and its effective management.

**Clinical trial number:**

Not applicable.

**Supplementary Information:**

The online version contains supplementary material available at 10.1186/s12909-025-06645-6.

## Background

Dentistry, as all medical disciplines, involves many uncertain situations and issues [1], and this has implications in the context of education. A challenge faced by educators is thus to familiarise students with sources of uncertainty and to make them comfortable with managing cases involving uncertainty. In theory, most clinical educators are aware that uncertainty exists in dentistry but in practice, clinicians as individuals may be more or less prone to acknowledge uncertainty in relation to practice [2]. Well-known situations where a dentist needs to make a judgement on uncertain basis is when assessing the risk for the patient to develop disease, for their disease to progress, or for symptoms to develop in an asymptomatic patient, such as exacerbation of apical periodontitis associated with a root filled tooth [1]. Other situations where most dentists would admit that it is possible to perceive uncertainty is for example when trying to determine the best path forward managing a tooth with radiographic evidence of approximal caries reaching past the enamel-dentin border and no previous images available, or when managing a tooth with a crack extending from a restoration towards the gingival sulcus. Uncertainty can be related to the clinician´s own lack of competence – the clinician simply does not have the knowledge that could be expected, and this type of uncertainty can be addressed by consulting various sources of knowledge. Other, perhaps more challenging aspects of uncertainty can be e.g., lack of scientific evidence such as about the ability to accurately identify dentin caries [3] or apical periodontitis in a radiograph [4], about the nature of the complex and dynamic interplay between the microorganisms and the host defense [5], and about the frequency of painful exacerbations and spread of infection from root filled teeth [6]. Uncertainty often has a negative effect on a person’s affective state, such as inducing a feeling of discomfort, but strategies to increase tolerance to uncertainty, and regulate emotional response can reduce the negative effect [7, 8]. Very few studies have investigated specifically how dentists manage uncertainty, but we believe that dental students would benefit from being introduced to such strategies.

With experience comes comfort with decisions. An experienced clinician is able to remain uncertain at the same time as taking action with confidence [9, 10], but to a clinician in their early career anxiety and discomfort may result from not knowing if the source of uncertainty is due to lack of scientific knowledge, or due to personal lack of knowledge [11]. Therefore, uncertainty and its sources should be discussed with students, and practical strategies for its management indicated to prepare them for future independent practice. Ilgen et al. defined different contributors of uncertainty as finding the situation ambiguous, perceiving the information as limited or recognizing that one has incomplete information [9]. To learn how to manage cases under conditions of uncertainty, students or early-career clinicians can practice using what Schön [12] called “reflection in action”. Various approaches to train students in reflection have been introduced in dental education [13–15]. Bain et al. [16]. developed a framework to help students in teachers’ education write reflections on their own practice. The framework was further developed by Ryan and Ryan [17] into the “4Rs of Reflection” model (Reporting & responding, Relating, Reasoning and Reconstructing). The goal with structured reflection according to the 4Rs is to make sense of experience and to reimagine future experience. The 4Rs provide a scaffold for the students while writing reflections by providing carefully constructed prompts which are simple for the student to follow and easy to remember. While following the prompts the reflection becomes deeper in a stepwise manner. Without scaffold it is unlikely that the students will reach critical reflection leading to deep learning or critical thinking [17].

The model aims to stimulate increasing depth of reflection and has been used across different disciplines in higher education such as fashion design [18], dance [19], psychology [20], law [21], accountancy [22], and social work practice [23]. In dental education the 4Rs were used in a study on how students’ reflections develop as they go through the dental education. A selected number of reflective texts written by students were analyzed and used to develop a five-aspect framework which included all prompts from the 4Rs [24].

Reflection as a means to deal with uncertainty has been studied in medical education where self-reflection in diaries was suggested to have aided medical students to develop a better tolerance of uncertainty [25], but no studies have specifically investigated the effects on dental students. The 4Rs were reported to help organize and deepen psychology students’ reflections [20], but we could not identify any publication from any discipline examining whether using the 4Rs can affect students´ awareness of and comfort with uncertainty.

## Methods

### Aim

The aims of the present study were (i) to measure the students’ comfort or discomfort with and awareness of various categories of uncertainty, as defined by Ilgen et al. [9], while conducting risk assessment and (ii) to investigate whether a reflection exercise would make the students more aware of, and comfortable with, uncertainty.

### Design

**Fig. 1:**
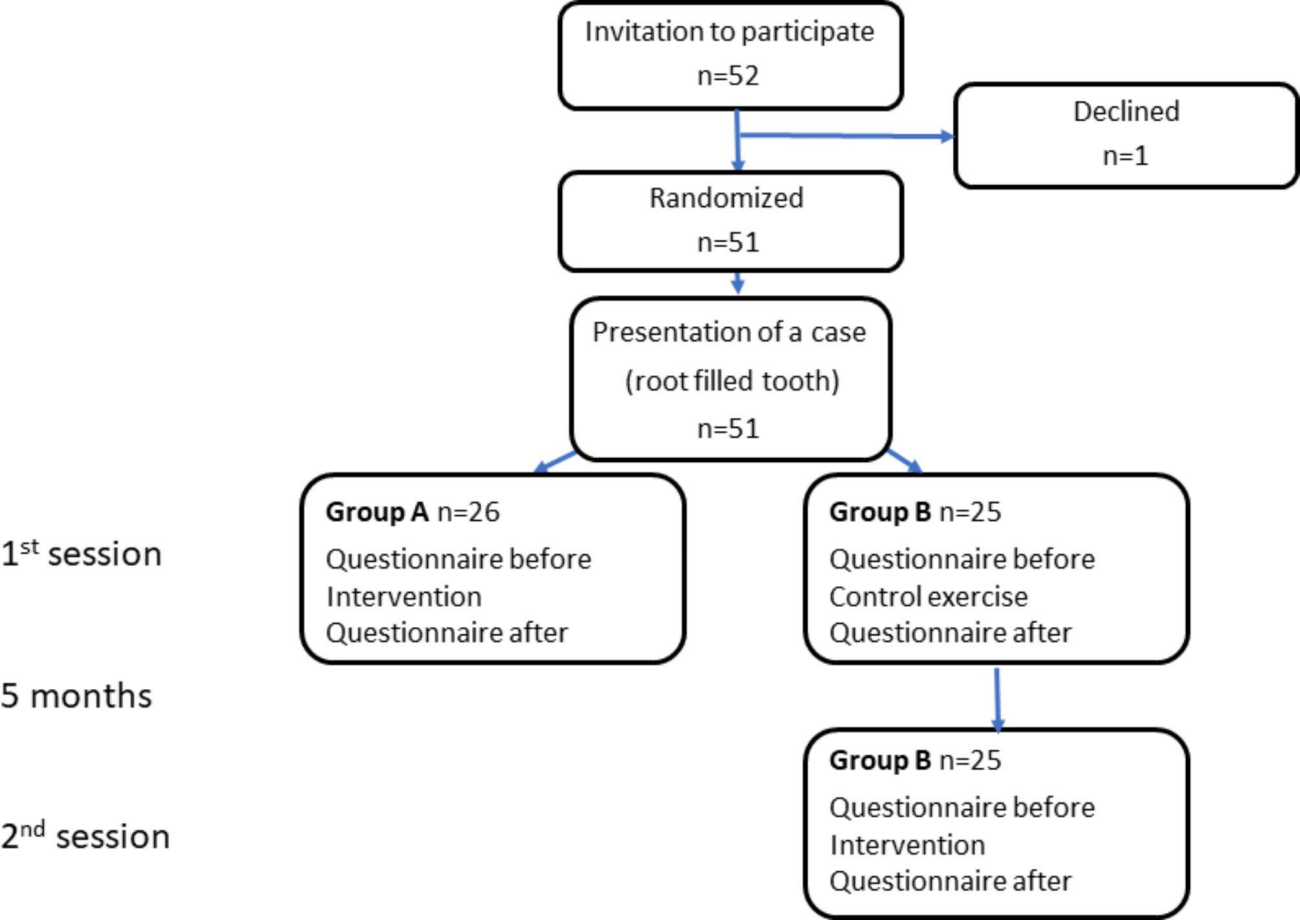
Experimental design with two student groups, Group A and Group B

This pretest-posttest study included a patient case with a radiograph, a questionnaire, an intervention in form of a reflection exercise, and a control exercise. The participants were randomized into two groups. The randomization was carried out in MS Excel (Microsoft Excel, version 2020 [Build 16.0.12345.67890], Microsoft Corporation, Redmond, WA, USA) and students were assigned to one group or the other. Students were blinded to whether they participated in the intervention or in the control exercise. They were assigned to one of the two exercises on the online learning platform Canvas (Instructure, Inc., Salt Lake City, UT, USA) and could not see the other exercise. In the first session, Group A were exposed to a reflection exercise (intervention) and Group B were exposed to the control exercise (control). In the second session five months later, Group B were exposed to the intervention (Fig. 1) and Group A to the control exercise. Both groups completed a questionnaire with questions about certainty and comfort with uncertainty related to the case. Both groups completed the questionnaire a total of four times; before and after each of the two exercises; but for group A, having been exposed to the intervention in January and therefore possibly not aligned with group B at baseline in June, only the results from January were analyzed (Fig. 1). The study followed the recommended reporting of experimental studies from Cook et al. [26].

### Participants and setting

As a part of the final year courses in 2021, all dental students (*n* = 52) in their 5th year at Malmö University, Sweden, were requested to participate in two mandatory internet-based sessions carried out on Canvas. The two sessions took place at the end of the two last semesters of the programme. These sessions formed the basis of the present study. One student participated in the sessions but did not consent to participate in the study. Their data was excluded, and the study thus included 51 participants (Fig. 1). At the time of the study, the students were attending theoretical courses and undergoing comprehensive clinical training in general dentistry, having completed their endodontic courses (theory, skills lab and patient treatment in the endodontic department) during their 3rd and 4th years. The students assigned to the intervention in Session 1 (Group A) were 18 women and 8 men, age ranging from 23 to 40 years (mean 28.0 ± SD 4.5) and the students exposed to the intervention in Session 2 (Group B) were 19 women and 6 men, age ranging from 24 to 41 years (mean 28.5 ± SD 5.3). The students did not receive information about the nature or purpose of the two exercises prior to participating in the study.

### Patient case

Before participating in one of the exercises in Canvas, all students were first presented with a patient case and a radiograph (Fig. 1). The case was a brief description of a patient with a root filled molar tooth without symptoms. Information about when the root filling was performed was not provided and was stated to be unavailable. The intraoral periapical radiograph showed a root filled tooth with diffuse widening of the periodontal ligament space and a large composite restoration. After reviewing the case, both groups were asked to complete a questionnaire with questions related to the case (Fig. 1).

### Questionnaire

Before answering the questions in the questionnaire, the participants were asked to estimate the probability of exacerbation of apical periodontitis within four years in relation to the connected case. They were asked: How likely do you think it is that the patient will experience an exacerbation of apical periodontitis within 4 years? The following questionnaire comprised six questions developed for the study (Table 1). All questions were developed from the theory of Ilgen et al. who conducted an extensive literature review and defined the concept of ‘comfort with uncertainty’ in clinical settings [9]. The first three questions (Q1–Q3) addressed the students’ feelings of certainty or uncertainty and comfort or discomfort in relation to the risk assessment. The final three questions (Q4–Q6) focused on whether the perceived uncertainty would fall into one of the three sources described by Ilgen et al. [9]; (i) perceiving limitations in one’s own knowledge, (ii) recognizing the situation as ambiguous, or (iii) recognizing the information as incomplete. A 5-point Likert-type scale was used, with response options ranging from *very certain/comfortable* to *very uncertain/uncomfortable*. The questionnaire was pilot tested prior to its publication on the online platform. The pilot involved two junior colleagues who had recently graduated as dentists and who gave feedback on the clarity of the questions. The questions were adjusted based on the feedback.

**Table 1:** Questionnaire developed for the study with questions related to various categories uncertainty and comfort or discomfort with uncertainty, as defined by Ilgen et al. [9]. Translated from Swedish

Q1	How certain do you feel about your assessment of the probability of the patient experiencing an exacerbation of apical periodontitis within 4 years?
Q2	How certain do you feel about your own capacity to handle this case?
Q3	How comfortable are you with your ability to handle the situation and do the best for the patient?
Q4	How certain do you think an experienced colleague would feel about assessing the probability of exacerbation of apical periodontitis within 4 years?
Q5	How certain do you feel that what you see on the radiograph is a sign of disease?
Q6	If you had access to more information about the patient (such as additional radiographs or examination findings, and relevant literature on the subject), how certain would you then feel about assessing the probability of the patient experiencing an exacerbation of apical periodontitis within 4 years?

### Intervention – reflection exercise

The intervention was a reflection exercise with seven questions adapted from prompts of the reflective scale by Ryan and Ryan [17] (Table 2). All the questions were related to the case that had been presented to the participants earlier. The questions were carefully constructed to encourage the students to think about the current case in relation to previous cases they had encountered and to theoretical reasoning from the literature. To encourage the students to reflect deeply, it was emphasized that they should describe the case from a biological perspective.

They were asked to write answers of at least 80 words to each question. They were informed that their contribution would not be graded, and they were asked to give honest answers. There were no time limits, and each question was locked after answering so that they could not go back and check or change the answers. Following the stepwise structure (progressing from superficial to deep reflection) of the 4Rs of Reflection model [17], the students were first asked to *report* about the case from a biologic point of view, and to *respond* by describing their strategy when assessing the risk for exacerbation of apical periodontitis in root filled teeth. Next, they were asked to *relate* the case to their own previous experiences; the students were asked about their knowledge and to recall a similar case that they had read about or encountered. They were also asked to explain what they needed to know more about. Subsequently, to help the students to *reason* about significant factors in the case, they were asked to think through the biological process and to fill in keywords to describe the biologic process based on their theoretical knowledge. To highlight the different sources of uncertainty and their own strengths or weaknesses, the students were also asked how they thought an expert would reason and if and how that would be different from their own way of reasoning. Finally, to help the students to *reconstruct* the problem they were asked to reflect on how they could reframe it if their chosen strategy for assessing the risk would turn out to be wrong, and to reflect on what could make them change strategy. Both groups were exposed to the intervention; Group A in January and group B in June (Fig. 1).

**Table 2:** The prompts for the reflection exercise used as the intervention. Adapted from a reflective scale by Ryan and Ryan [17]. Translated from Swedish

1	What is the case about? Describe the case from a biological point of view.
2	What strategy do you have when assessing the probability for exacerbation of apical periodontitis in this case?
3	What knowledge do you have about assessing the probability of exacerbation of apical periodontitis in a case like this? Think of a similar case that you have read about or encountered before. Is there any factor that is different in this case that could indicate a different biological condition? Explain!
4	What are the risks for the patient? What do you really know about the risk? What do you need to know more about? Explain!
5	Think through the biological process, based on theory, and fill in keywords describing biological factors from a leaking restoration to the exacerbation of apical periodontitis.
6	How do you think an expert would reason about the patient’s condition and assess the risk? Is it different from your way of reasoning?
7	If your chosen strategy turns out to be wrong – it could mean either that you overtreated the patient, i.e., that you treated a condition that did not require treatment, or that you neglected to treat a condition that required treatment, i.e., undertreatment. Think about what is best from the perspective of efficient utilization of resources and from the ethical perspective. Reflect on what could make you change strategy.

### Control exercise

The control exercise was a quiz comprising 13 multiple-choice questions (MCQ) about instrumentation during endodontic treatment. The questions had low level of difficulty considering the students’ current level of training and did not intend to stimulate reflection, and the students were given no prompts for reflection (Fig. 1).

### Data analysis

Statistical analysis was performed using IBM SPSS Statistics version 25.0 (SPSS Inc, Chicago, IL, USA). Mann-Whitney U-tests were performed to compare the questionnaire responses following the intervention and the control exercise, respectively. The tests were performed separately for the 1st and 2nd time the students completed the questionnaire. To reveal possible changes in the responses to the questionnaire within the groups from the 1st time to the 2nd time for both groups and additionally from the 3rd time to the 4th time for group B, the responses were compared performing Wilcoxon signed rank test. Differences were considered statistically significant at the *p* < 0.05 level.

## Results

### Participation and response rate

In both sessions, all 51 students completed the questionnaire with a response rate of 100%.

### Effect of the reflection exercise

At baseline, when the questionnaire was completed the 1st time, there were no differences in the distribution of the responses to any of the questions between the students in Group A and Group B (*p* = 0.210–0.871). After the exercises of Session 1 when the questionnaire was completed the 2nd time there were still no differences between the groups (*p* = 0.107–0.496; Additional file 1, Table 1).

**Fig. 2:**
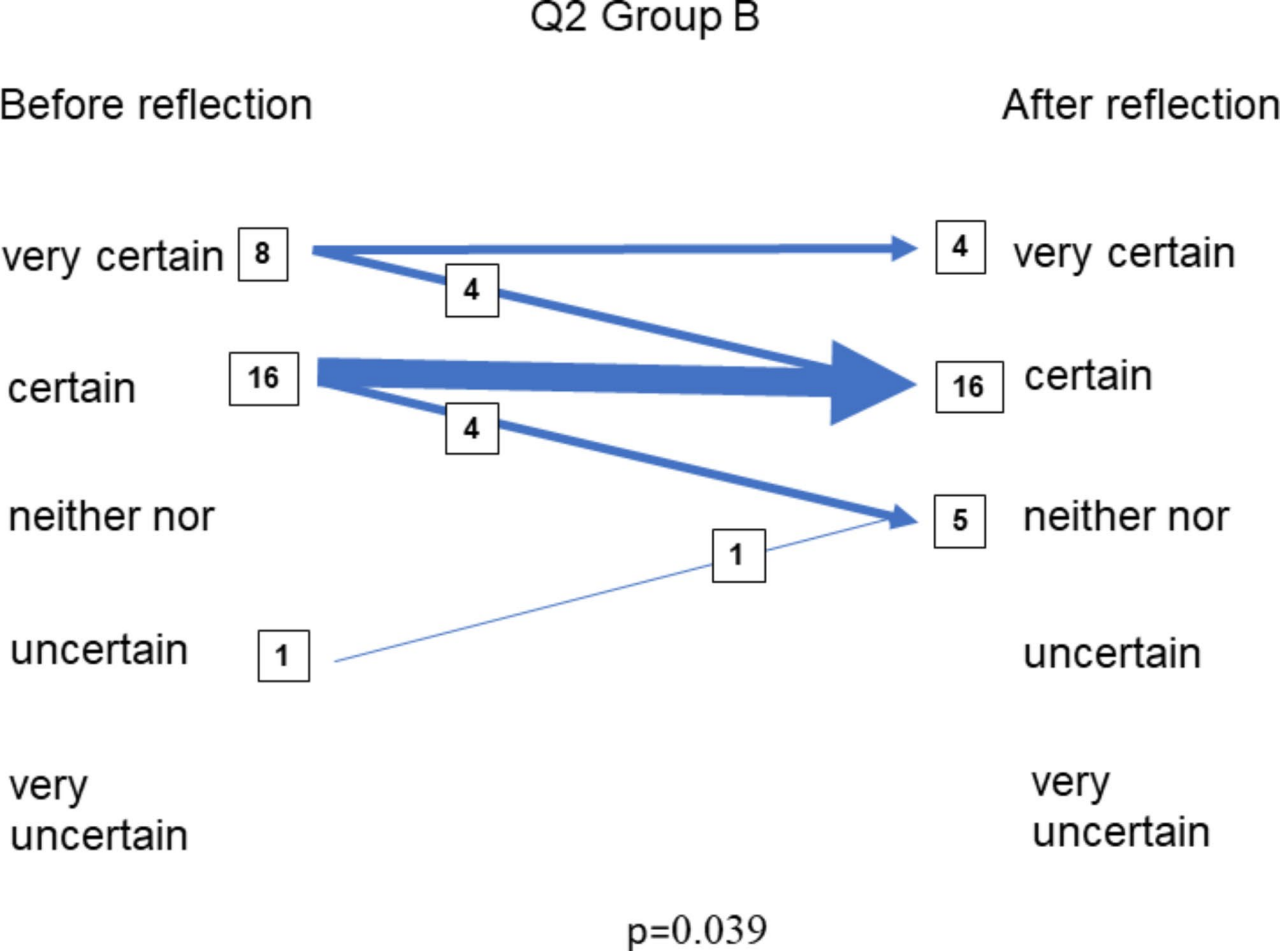
The figure shows the number of students who changed their response (arrow thickness) and how their responses were changed (arrow direction) in regard to Q2

In June when the students in Group B, were exposed to in the reflection exercise, there was a difference between the distribution of the responses within the group before the intervention and after the intervention to one question, Q2 (*How certain do you feel about your own capacity to handle this case?p* = 0.039) (Fig. 2).

### Awareness of different types of uncertainty

#### Perceiving limitations in one’s own knowledge

At baseline, before Session 1, 80% (41/51) of the students in both groups, stated feeling very uncertain (*n* = 4), uncertain (*n* = 12) or neither certain nor uncertain (*n* = 25) and 20% (10/51) stated feeling certain to Q1 (*How certain do you feel about your assessment of the probability of the patient experiencing an exacerbation of apical periodontitis within 4 years?*). There was no difference within any group after the intervention or the control exercise (Additional file 1, Table 1). To Q4 (*How certain do you think an experienced colleague would feel about assessing the probability of exacerbation of apical periodontitis within 4 years?*) the majority of the students, 57% (29/51), stated that they thought an experienced colleague would feel very certain (*n* = 2) or certain (*n* = 27) and 43% (22/51) neither certain nor uncertain (*n* = 20) or uncertain (*n* = 2).

#### Recognizing the situation as ambiguous and recognizing the information as incomplete

There was no difference between the groups at baseline to Q5 (*How certain do you feel that what you see on the radiograph is a sign of disease?*) and Q6 (*If you had access to more information about the patient [such as additional radiographs or examination findings*,* and relevant literature on the subject] how certain would you then feel about assessing the probability of the patient experiencing an exacerbation of apical periodontitis within 4 years?*). After the intervention and the control session there was still no difference between the groups or within any group (Table 1).

#### The comfort with uncertainty

At baseline, 84% (43/51) of the students stated feeling *very comfortable* (*n* = 15) or *comfortable* (*n* = 28) in regard to Q3 (*How comfortable are you with your ability to handle the situation and do the best for the patient?*), while 10% (5/51) stated feeling *neither comfortable nor uncomfortable*, and 6% (3/51) stated feeling *uncomfortable*. There was no difference between the groups or within any group after the intervention and the control exercise.

## Discussion

In this study, a reflection exercise targeting dental students was designed. At baseline, only 20% of the students stated that they felt certain about assessing the risk for exacerbation of apical periodontitis yet despite not feeling certain about assessing the risk, 80% stated feeling certain or very certain about their capacity to handle the case and 84% stated feeling comfortable or very comfortable with their ability to handle the situation. Since a large proportion of the students already felt comfortable before the exercise, the exercise did not have great potential to increase the comfort with uncertainty as tentatively intended a priori.

### The intervention

According to Dewey [27], reflection occurs in uncertain, doubtful or problematic situations and the process of reflection is a way of learning from experience. In the present study, reflection was used as a method to explore and reveal different sources of uncertainty. The students were presented with a case which intended to resemble patients that they could encounter at their clinical practice. The influence of reflection on the students’ awareness of different sources of uncertainty and their comfort with uncertainty was investigated by comparing the students’ responses to a questionnaire before and after the exercise. The assumption underlying the experimental design was that after reflecting on their own previous experience with similar cases and on general related topics – for example the biological risk factors for exacerbation of apical periodontitis and the probability of such an event occurring – the students would become more aware of different sources of uncertainty and that this awareness would also affect their feeling of comfort during risk assessment. In contrast, the assumption was that the control exercise was designed not to stimulate reflection and would thus not affect the students’ awareness of the sources of uncertainty and their comfort with uncertainty. These assumptions could however not be corroborated within this study. There were no differences in questionnaire results between the groups before and after the intervention and the control exercise, which may indicate that the intervention did not have any direct effect on the awareness of uncertainty.

### The experimental design

A crossover design was chosen to ensure sufficient sample size while also including study participants with as close to the same background exposure as possible, i.e. students from the same teaching cohort. On each occasion, the students were not informed of the purpose of the exercises and were blinded to the type of session they were randomized to. The students in group A participated in the control exercise in June but the results from the questionnaire were not analyzed since they had already participated in the intervention in January, which could conceivably bias the “effect” of the control exercise by the students already being more prone to reflect on the (identical) task. This way the blinding was kept throughout the study. For this reason, the students did not receive an introduction to professional reflection before participating in the intervention, despite that it has been advocated that students should be introduced to the concept of professional reflection before they are asked to reflect [28]. However, by providing the students with prompts from the 4Rs, the students were given a scaffold to help them write down their reflections in a structured way. A follow-up discussion of the intervention with the student group, a reflection-on-action [12], might have furthered the students’ understanding of different sources of uncertainty and how they manage these uncertainties in their decision-making. However, the crossover setup where the other student group would participate in the intervention in the second session, five months later, did not allow for this. An additional potential bias may be that a short time before each of the two sessions, the students sat their examinations in which one assignment was centered around a case with a patient experiencing exacerbation of apical periodontitis in a root filled tooth. The questions of the exam covered risk assessment, aetiology and treatment considerations, and it is possible that this stimulated some reflection on the issue also in the students assigned to the control exercise in the corresponding session shortly afterwards. Other than this, there were no specific educational activities targeting assessment of the risk for exacerbation of apical periodontitis associated with root filled teeth and the sources of uncertainty, but between the two sessions all students handled patients in the comprehensive dentistry clinic and some of them may have encountered exacerbations or been engaged in discussions related to risk assessment in similar situations with peers and teachers. In addition, it cannot be excluded that students might also have discussed the exercises of this study between themselves after the first session, and therefore the results were not compared between the student groups participating in the reflection exercise in January and in June. Overall, the longitudinal crossover design has potential to introduce bias, and efforts were made to mitigate the effects of any activities between the two sessions that may have affected the outcome.

### The lack of uncertainty

Unexpectedly, the majority of students stated that they felt certain of their ability to handle the case properly even if they did not feel certain about assessing the risk. One student explained, in a written reflection during the intervention: *“Most cases of apical periodontitis are asymptomatic and a large percentage (about 30% I think) of root-filled teeth show apical periodontitis. It is thus not a lack of symptoms that guides the choice to revise a root-filling or not. In order to assess the likelihood of exacerbation*,* I need to decide whether what I see on the X-ray is really pathology or whether it is healing. The case above could be a picture of healing. The lesion may have shrunk from its original size or connective tissue healing has occurred. In both of these cases*,* the likelihood of exacerbation is low as there is no root canal infection and there is no pathological bone breakdown.”*

Another student wrote: “*If the patient is in good health with no risk factors and I am uncertain of my assessment*,* I use a strategy to undertreat (wait and see)*,* but the downside is that the patient may have problems in the future*,* so I inform the patient of this.”*

The students’ lack of uncertainty was surprising since there is a lack of scientific evidence about risks, e.g. the probability of exacerbation of apical periodontitis associated with a root filled tooth, which makes it impossible for anyone to be certain about the best way to manage the case [1]. One hypothesis to explain this apparent conflict is that dental students are taught to be certain. The role of educators appears crucial, both on the curriculum level (learning outcomes and examination forms) and in theoretical, preclinical and clinical teaching. Von Bergmann and Shuler wrote in a recent article that the dental education has too much of a culture of certainty: “Our learning environments, as well as our professional environments, need to begin accepting that a dental student or a dental professional can safely admit that they do not know and can treat uncertainty as an opportunity to learn” [29]. Furthermore, it was suggested that time concerns, e.g. the perceived need of the professional to make decisions and act without delay, threaten practice of critical thinking, such as questioning one’s judgement and thinking of other possible explanations and differential diagnoses [29]. A study on educators’ impact on how medical students deal with uncertainty by Bochatay & Bajwa [30] compared students in two different medical centers and found that the supervisors’ attitudes affected how students learned to manage uncertainty. In one of the centers, where the supervisors treated the residents’ expressed uncertainty as learning opportunities, the students developed an accepting attitude towards uncertainty. In contrast, in the other medical center where the supervisors provided additional instructions in response to the students’ expressed uncertainty but did not engage in discussion, the students developed a pragmatic attitude towards uncertainty as a matter to be addressed and controlled. The results from the present study suggest that the students believe that certainty comes with experience. They stated that they believed that an experienced colleague, perhaps a clinical supervisor, would feel certain when assessing the risk for exacerbation of apical periodontitis. One student wrote: “*What differs from my way*,* I believe*,* has a lot to do with experience*,* that an expert has seen a large number of similar cases and can therefore assess the risk faster and with greater certainty. An expert*,* I think*,* has a much better idea about cost-effectiveness and in which specific cases the benefit of treatment can exceed the cost in the form of treatment risk for the patient and also financially for the healthcare and the patient.*” The combination “lack of uncertainty” and “lack of evidence” is interesting and should be further explored.

### Experience and the perception of certainty

Only 4% of all students stated at baseline that they believed that an experienced colleague would feel uncertain, and we speculate that this may reflect the teaching approach by the clinical supervisors. This is an interesting finding that merits further investigation and possibly intervention, because the literature suggests that rather than act as if always confident, teachers should acknowledge their uncertainty and invite discussion with students to help them develop into reflecting practitioners who are aware of and comfortable with uncertainty and prepared to act despite it [29–31].

Experience does not equal certainty. Again citing von Bergmann and Shuler: “It is critical for the profession as a whole to recognize that there is nothing wrong with a clinician being uncertain about a diagnosis/…/Recognizing the potential for uncertainty in any clinical situation is an important clinical skill and one that the most experienced clinicians have dealt with repeatedly.”

Further studies exploring dentistry teachers’ awareness, attitudes and teaching strategies related to uncertainty are ongoing, but one recommended strategy, based on previous research, would be to encourage clinical instructors to be open with their own feelings of uncertainty and discuss with students how to manage in such situations [29, 30].

### The sources of uncertainty

Approximately half of the students in the present study did not state that they felt certain that what they saw in the radiograph was a sign of disease, indicating that these students acknowledged the ambiguity of the radiographic findings [32]. None of the students stated that they would feel uncertain with access to more information, which appears reasonable since the information in the case was very limited; in particular, no information was given about the treatment history of the patient. The purpose with providing limited information was to stimulate reflection and to mimic a real case with a new patient (where available information on history can be scarce). The interpretation of this finding is that all students were able to correctly identify the lack of information as a source of uncertainty.

When students (and clinicians) assess risk, such as in this study the risk for exacerbation in a specific case featuring a root filled tooth with apical periodontitis, they should be able to consciously distinguish between various sources of uncertainty, these being a personal lack of knowledge and experience, a lack of case-specific useful information or ambiguity of findings, and a more general lack of scientific knowledge regarding the issue. The reason for this is that for some sources, the uncertainty can be reduced (by consulting with a colleague or relevant literature or by collecting more clinical information) but for other sources, uncertainty must be accepted, at least until more research is available. This study was limited to examining risk assessment related to persistent apical periodontitis associated with root-filled teeth, but in a wider perspective it can be seen as an example or model condition relevant to management of uncertainty and can be applied in other areas of dental education or indeed also other medical specialties.

### Future research

Assessing subtle constructs like uncertainty is challenging. Several dimensions are discernable, which may need to be addressed in both quantitative and qualitative studies. Future research could focus on studying both educators and dental students and how they cope with uncertainty in dentistry, especially when assessing risk, which is closely related to assessment of prognosis. An associated question might be to study, with a qualitative approach, how clinical instructors’ attitudes to uncertainty are reflected in their teaching. The written reflections from the intervention in the present study will also be analyzed to examine if the students really did engage in complex professional reflection. As proposed by the 4Rs model, achieving this requires combining previous clinical experience with knowledge of ethics and science to reimagine and reflect on how to improve [33].

## Strengths and limitations

A strength with the study was the crossover setup and that the students were blinded to which of the exercises was the intervention. This ensured that the thought of being in a study did not have different effects on the intervention and the control exercise. A limitation was that the questions in the questionnaire were developed for this study, since no validated instrument suitable for the aims of this study could be identified. Using a validated questionnaire would perhaps have strengthened the results in that the results could be compared to those of other studies. However, to achieve the aim, to measure the students’ awareness of and comfort with uncertainty while conducting risk assessments, and to investigate whether the reflection exercise would increase their awareness and comfort with uncertainty, the questions needed to be very specific and closely follow the theory by Ilgen et al. [9] which describes various aspects of ‘comfort with uncertainty’ in clinical settings. This theory provided us with a detailed framework to develop the questions.

Other limitations are the modest sample size and the fact that the study was carried out in a single institution and therefore may have limited generalizability to other settings. The study is exploratory and could be repeated with a larger and more diverse sample, thereby also possibly validating the developed questionnaire.

## Conclusion

With the short reflection exercise developed for the study we were able to measure the students’ awareness of uncertainty and their feeling of comfort with it. However, the exercise did not affect the feeling of comfort with uncertainty since the majority of the students stated feeling comfortable with their capacity to handle the situation already before the intervention. Although the reflection exercise did not directly increase the students’ comfort with uncertainty, it highlighted the students’ perception that experience plays a significant role in managing uncertainty.

## Electronic supplementary material

Below is the link to the electronic supplementary material.

Supplementary Material 1

## Data Availability

Data is provided within the manuscript or supplementary information files.
