# Supplementary material for: Reflection to enhance dental students´ awareness of and comfort with uncertainty – an experimental study
Source: BMC Med Educ. 2025 Jan 10;25:46. doi: 10.1186/s12909-025-06645-6 (PMC11724501; doi:10.1186/s12909-025-06645-6)
Supplement: Supplementary file 1 — Supplementary Material 1 [file 12909_2025_6645_MOESM1_ESM.docx]

**Additional file.** Table 1, Questionnaire questions with response alternatives before and after the exercises in January. Results of the student groups responses and comparison between groups using Mann-Whitney U-tests. Differences with p<0.05 would have been considered statistically significant.

|  |  | Questionnaire before | | | Questionnaire after | | | |
| --- | --- | --- | --- | --- | --- | --- | --- | --- |
| # Question | Alternative | Group A (n=26) | Group B (n=25) | p -value | | Group A (n=26) | Group B (n=25) | p-value |
| 1 | very certain | 0 | 0 |  | | 0 | 0 |  |
|  | certain | 5 | 5 |  | | 8 | 4 |  |
|  | neither nor | 16 | 9 | 0.244 | | 14 | 13 | 0.107 |
|  | uncertain | 3 | 9 |  | | 4 | 7 |  |
|  | very uncertain | 2 | 2 |  | | 0 | 1 |  |
| 2 | very certain | 6 | 7 |  | | 6 | 5 |  |
|  | certain | 17 | 11 |  | | 15 | 12 |  |
|  | neither nor | 1 | 4 | 0.650 | | 4 | 8 | 0.467 |
|  | uncertain | 2 | 3 |  | | 1 | 0 |  |
|  | very uncertain | 0 | 0 |  | | 0 | 0 |  |
| 3 | very comfortable | 8 | 7 |  | | 4 | 6 |  |
|  | comfortable | 13 | 15 |  | | 15 | 14 |  |
|  | neither nor | 2 | 3 | 0.758 | | 7 | 5 | 0.496 |
|  | uncomfortable | 3 | 0 |  | | 0 | 0 |  |
|  | very uncomfortable | 0 | 0 |  | | 0 | 0 |  |
| 4 | very certain | 2 | 0 |  | | 4 | 2 |  |
|  | certain | 12 | 15 |  | | 14 | 13 |  |
|  | neither nor | 12 | 8 | 0.871 | | 8 | 8 | 0.305 |
|  | uncertain | 0 | 2 |  | | 0 | 2 |  |
|  | very uncertain | 0 | 0 |  | | 0 | 0 |  |
| 5 | very certain | 2 | 3 |  | | 2 | 1 |  |
|  | certain | 12 | 6 |  | | 11 | 8 |  |
|  | neither nor | 3 | 10 | 0.819 | | 8 | 11 | 0.426 |
|  | uncertain | 7 | 4 |  | | 3 | 3 |  |
|  | very uncertain | 2 | 2 |  | | 2 | 2 |  |
| 6 | very certain | 9 | 5 |  | | 8 | 3 |  |
|  | certain | 13 | 13 |  | | 14 | 16 |  |
|  | neither nor | 4 | 7 | 0.210 | | 4 | 5 | 0.108 |
|  | uncertain | 0 | 0 |  | | 0 | 1 |  |
|  | very uncertain | 0 | 0 |  | | 0 | 0 |  |
